# RawHash2: mapping raw nanopore signals using hash-based seeding and adaptive quantization

**DOI:** 10.1093/bioinformatics/btae478

**Published:** 2024-07-30

**Authors:** Can Firtina, Melina Soysal, Joël Lindegger, Onur Mutlu

**Affiliations:** Department of Information Technology and Electrical Engineering, ETH Zurich, Zurich 8092, Switzerland; Department of Information Technology and Electrical Engineering, ETH Zurich, Zurich 8092, Switzerland; Department of Information Technology and Electrical Engineering, ETH Zurich, Zurich 8092, Switzerland; Department of Information Technology and Electrical Engineering, ETH Zurich, Zurich 8092, Switzerland

## Abstract

**Summary:**

Raw nanopore signals can be analyzed while they are being generated, a process known as real-time analysis. Real-time analysis of raw signals is essential to utilize the unique features that nanopore sequencing provides, enabling the early stopping of the sequencing of a read or the entire sequencing run based on the analysis. The state-of-the-art mechanism, RawHash, offers the first hash-based efficient and accurate similarity identification between raw signals and a reference genome by quickly matching their hash values. In this work, we introduce RawHash2, which provides major improvements over RawHash, including more sensitive quantization and chaining algorithms, weighted mapping decisions, frequency filters to reduce ambiguous seed hits, minimizers for hash-based sketching, and support for the R10.4 flow cell version and POD5 and SLOW5 file formats. Compared to RawHash, RawHash2 provides better F1 accuracy (on average by 10.57% and up to 20.25%) and better throughput (on average by 4.0× and up to 9.9×) than RawHash.

**Availability and implementation:**

RawHash2 is available at https://github.com/CMU-SAFARI/RawHash. We also provide the scripts to fully reproduce our results on our GitHub page.

## 1 Introduction

Nanopore technology can sequence long nucleic acid molecules up to more than two million bases at high throughput (Jain *et al.* 2018). As a molecule moves through a tiny pore, called a *nanopore*, ionic current measurements are generated at a certain throughput [e.g., around 450 bases per second for DNA (Kovaka *et al.* 2021, Zhang *et al.* 2021)]. These electrical measurements, known as *raw signals*, can be used to (i) identify individual bases in the molecule with computational techniques such as *basecalling* (Senol Cali *et al.* 2019) and (ii) analyze raw signals directly *without* translating them to bases (Firtina *et al.* 2023a).

**Figure 1.**
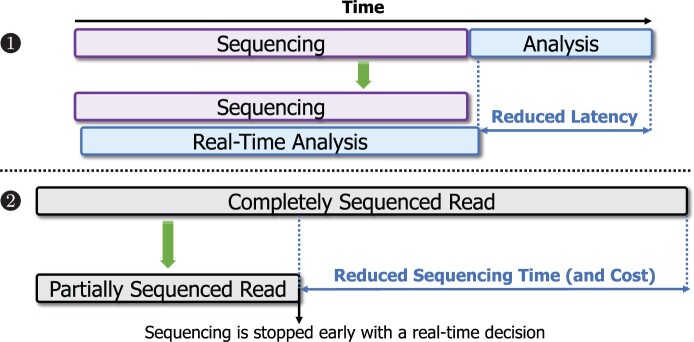
Two main benefits of real-time analysis with nanopore sequencing.

Computational techniques that can analyze the raw signals while they are generated at a speed that matches the throughput of nanopore sequencing are called *real-time analysis*. Figure 1 shows the two unique benefits that real-time analysis offers. First, real-time analysis allows for overlapping sequencing time with analysis time, as raw signals can be analyzed while they are being generated. Second, computational mechanisms can stop the sequencing of a read or the entire sequencing run early without sequencing the entire molecule or the sample using techniques known as Read Until (Loose *et al.* 2016) and Run Until (Payne *et al.* 2021). The development of accurate and fast mechanisms for real-time analysis has the potential to significantly reduce the time and cost of genome analysis.

There are several mechanisms that can perform real-time analysis of raw nanopore signals to achieve accurate and fast genome analysis (Edwards *et al.* 2019, Bao *et al.* 2021, Dunn *et al.* 2021, Kovaka *et al.* 2021, Payne *et al.* 2021, Zhang *et al.* 2021, Firtina *et al.* 2023a, Lindegger *et al.* 2023, Mikalsen and Zola 2023, Sadasivan *et al.* 2023, Shih *et al.* 2023, Shivakumar *et al.* 2023). Most of these solutions have three main limitations. First, many mechanisms offer limited scalability or support on resource-constrained devices due to their reliance on either (i) deep neural networks (DNNs) for real-time base translation, which are usually computationally intensive and power-hungry (Payne *et al.* 2021, Ulrich *et al.* 2022), or (ii) specialized hardware such as ASICs or FPGAs (Bao *et al.* 2021, Dunn *et al.* 2021, Shih *et al.* 2023). Second, while some mechanisms can directly analyze raw signals without base translation, offering an efficient alternative for real-time analysis (Kovaka *et al.* 2021, Zhang *et al.* 2021), they often compromise accuracy or performance when applied to larger genomes. Third, methods based on machine learning often require retraining or reconfiguration (Bao *et al.* 2021, Sadasivan *et al.* 2023, Senanayake *et al.* 2023), adding a layer of complexity and reducing their flexibility for general use cases, such as read mapping to any genome.

Among the existing works, RawHash (Firtina *et al.* 2023a) is the state-of-the-art mechanism that can accurately perform real-time mapping of raw nanopore signals for large genomes without translating them to bases with a hash-based seed-and-extend mechanism (Altschul *et al.* 1990). Despite its strengths in accuracy and performance, particularly for large genomes like the human genome, RawHash exhibits several limitations that require further improvements. First, RawHash utilizes a simple quantization algorithm that assumes the raw signals are distributed uniformly across their normalized value range, which limits its efficiency and accuracy. Second, RawHash uses a chaining algorithm similar to that used in Sigmap (Zhang *et al.* 2021) without incorporating penalty scores used in minimap2 (Li 2018), which constrains its ability for more sensitive mapping. Third, RawHash performs chaining on all seed hits without filtering any of these seed hits, which substantially increases the workload of the chaining algorithm due to a large number of seed hits to chain. Fourth, the decision-making mechanism in RawHash for mapping reads to a reference genome in real-time relies on one of the mapping conditions being true (e.g., the ratio between the best and second-best chain scores), which makes it more prone to the outliers that can satisfy one of these conditions. A more robust and statistical approach that incorporates features beyond chaining scores can provide additional insights for making more sensitive and quick mapping decisions. Fifth, while the hash-based mechanism in RawHash is compatible with existing sketching techniques such as minimizers (Roberts *et al.* 2004, Li 2018), strobemers (Sahlin 2021), and fuzzy seed matching as in BLEND (Firtina *et al.* 2023b), the benefits of these techniques are unknown for raw signal analysis as they are not used in RawHash. Such evaluations could potentially provide additional insights on how to use the existing hash-based sketching techniques and reduce storage requirements while maintaining high accuracy. Sixth, RawHash lacks the support for recent advancements, such as the newer R10.4 flow cell version. The integration of these features can accelerate the adoption of both real-time and offline analysis.

In this work, our goal is to address the aforementioned limitations of RawHash by improving its mechanism. To this end, we propose RawHash2 to improve RawHash in six directions. First, to generate more accurate and unique hash values, we introduce a new quantization technique, *adaptive quantization*. Second, to improve the accuracy of chaining and subsequently read mapping, we implement a more sophisticated chaining algorithm that incorporates penalty scores (as in minimap2). Third, to improve the performance of chaining by reducing its workload, RawHash2 provides a filter that removes seeds frequently appearing in the reference genome, known as a *frequency filter*. Fourth, we introduce a statistical method that utilizes multiple features for making mapping decisions based on their weighted scores to eliminate the need for manual and fixed conditions to make decisions. Fifth, we extend the hash-based mechanism to incorporate and evaluate the minimizer sketching technique, aiming to reduce storage requirements without significantly compromising accuracy. Sixth, we integrate support for R10.4 flow cells and more recent file formats, POD5 and S/BLOW5 (Gamaarachchi *et al.* 2022).

Compared to RawHash, our extensive evaluations on five genomes of varying sizes and six different real datasets show that RawHash2 provides higher accuracy (by 10.57% on average and 20.25% at maximum) and better read mapping throughput (by 4.0× on average and 9.9× at maximum). We make the following contributions:

- We propose substantial algorithmic improvements to the state-of-the-art tool, RawHash. These include (i) more accurate quantization, (ii) more sensitive chaining with penalty scores, (iii) a frequency filter, (iv) mapping decisions based on a weighted sum of several features that can contribute to the decision, (v) the minimizer sketching technique.
- We provide the support and evaluation for the newer flow cell version (i.e., R10.4) and file formats (i.e., POD5 and SLOW5).

## 2 Materials and methods

RawHash is a mechanism to perform mapping between raw signals by quickly matching their hash values. We provide the details of the RawHash mechanism in Supplementary Section A. RawHash2 provides substantial improvements over RawHash in six key directions. First, to generate more accurate and distinct hash values from raw signals, RawHash2 improves the quantization mechanism with an *adaptive* approach such that signal values are quantized nonuniformly based on the characteristics of a nanopore model. Second, to provide more accurate mapping, RawHash2 improves the chaining algorithm in RawHash with more accurate penalty scores. Third, to reduce the workload in chaining for improved performance, we integrate a frequency filter to quickly eliminate the seed hits that occur too frequently. Fourth, to make more accurate and quick mapping decisions, RawHash2 determines whether a read should be mapped at a specific point during sequencing by using a weighted sum of multiple features. Fifth, to reduce the storage requirements of seeds, RawHash2 incorporates and evaluates the benefits of minimizer sketching technique. Sixth, RawHash2 includes support for the latest features introduced by ONT, such as new file formats and flow cells.

### 2.1 Adaptive quantization

To improve the accuracy and uniqueness of hash values generated from raw nanopore signals, RawHash2 introduces a new *adaptive* quantization technique that we explain in four steps.

First, to enable a more balanced and accurate assignment of normalized signal values into quantized values (i.e., buckets), RawHash2 performs a bifurcated approach to define two different ranges: (i) fine range and (ii) coarse range. These ranges are useful for fine-tuning the boundaries of normalized signal values, *s* falling into a certain quantized value, *q*(*s*) within the integer value range [0, *n*], as the normalized distribution of signal values is not uniform across all ranges. Second, within the *fine range*, normalized signal values are quantized into smaller intervals to enable a high resolution, *f_r_*, for quantization due to the larger number of normalized signal values that can be observed within this range. The boundaries of the fine range, (*f*_min_ and *f*_max_), are empirically defined to enable robustness and high accuracy applicable given a flow cell (e.g., R9.4) and parameters to RawHash2 to enable flexibility. Third, the normalized signal values outside the fine range (i.e., the *coarse range*) are quantized into larger intervals with low resolution, $c_{r}=(1-f_{r})\times 0.5$, to enable a more balanced load of quantized values across all ranges by assigning more signal values within this range into the same quantized value. Fourth, depending on the range that a normalized signal is in, its corresponding quantized value is assigned as shown in Equation (1). The adaptive quantization approach can enable a more balanced and accurate distribution of quantized values by better distinguishing closeby signal values with high resolution and grouping signals more efficiently in the coarser range.

(1) $$q(s)=\{\begin{array}{cc} \left\lfloor n\times (f_{r}\times \frac{\left(s-f_{\min}\right)}{f_{\max}-f_{\min}}\right) & \text{if}\;f_{\min}\leq s\leq f_{\max}\\ \left\lfloor n\times (f_{r}+c_{r}\times s\right) & \text{if}\;s<f_{\min}\\ \left\lfloor n\times (f_{r}+c_{r}+c_{r}\times s\right) & \text{if}\;s>f_{\max} \end{array}$$

### 2.2 Chaining with penalty scores

To identify the similarities between a reference genome (i.e., target sequence) and a raw signal (i.e., query sequence), the series of seed hits within close proximity in terms of their matching positions are identified using a dynamic programming (DP) algorithm, known as *chaining*. Using a chaining terminology similar to that of minimap2 (Li 2018), a seed hit between a reference genome and a raw signal is usually represented by a 3-tuple (*x*, *y*, *w*) value, known as *anchor*, where *w* represents the length of the region that a seed spans, the start and end positions of a matching interval in a reference genome and a raw signal is represented by [x−w+1,x] and [y−w+1,y], respectively. The chain of anchors within close proximity is identified by calculating the optimal chain score *f*(*i*) of each anchor *i*, where *f*(*i*) is calculated based on predecessors of anchor *i* when anchors are sorted by their reference positions. To calculate the chain score, *f*(*i*), with dynamic programming, RawHash performs the following computation as used in Sigmap (Zhang *et al.* 2021).

(2) $$f(i)=\max\{\underset{i>j\geq 1}{\max}\{f(j)+\alpha (j,i)\},w_{i}\}$$

where $\alpha (j,i)=\min\{\min\{y_{i}-y_{j},x_{i}-x_{j}\},w_{i}\}$ is the length of the matching region between the two anchors. Although such a design is useful when identifying substantially fewer seed matches using a seeding technique based on distance calculation as used in Sigmap, RawHash identifies a larger number of seed matches as it uses hash values to identify the matching region, which is usually faster than a distance calculation with the cost of reduced sensitivity.

To identify the correct mapping regions among such a large number of seed matches, RawHash2 uses a more sensitive chaining technique as used in minimap2 by integrating the gap penalty scores such that the chain score of an anchor *i* is calculated as shown in Equation (3):

(3) $$f(i)=\max\{\underset{i>j\geq 1}{\max}\{f(j)+\alpha (j,i)-\beta (j,i)\},w_{i}\}$$

where $\beta (j,i)=\gamma_{c}((y_{i}-y_{j})-(x_{i}-x_{j}))$ is the penalty score calculated based on the gap distance, *l*, between a pair of anchors *i* and *j* where $\gamma_{c}(l)=0.01\cdot w\cdot |l|+0.5{\;\text{log}\;}_{2}|l|$. Based on the chain score calculation with gap costs, RawHash2 integrates similar heuristics, mapping quality calculation, and the same complexity when calculating the chaining scores with the gap penalty as described in minimap2 (Li 2018).

### 2.3 Frequency filters

RawHash2 introduces a two-step frequency filtering mechanism to (i) reduce the computational workload of the chaining process by limiting the number of anchors it processes and (ii) focus on more unique and potentially meaningful seed hits. First, to reduce the number of queries made to the hash table for identifying seed hits, RawHash2 eliminates nonunique hash values generated from raw signals that appear more frequently than a specified threshold. Second, RawHash2 evaluates the frequency of each seed hit within the reference genome and removes those that surpass a predefined frequency threshold, which reduces the overall workload of the chaining algorithm by providing a reduced set of more unique seed hits.

### 2.4 Weighted mapping decision

RawHash performs mapping while receiving chunks of signals in real-time, as provided by nanopore sequencers. It is essential to decide if a read maps to a reference genome as quickly as possible to avoid unnecessary sequencing. The decision-making process in RawHash is based on a series of conditional checks involving chain scores. These checks are performed in a certain order and against fixed ratios and mean values, making the decision mainly rigid and less adaptive to variations.

To use a more statistical approach that can generalize various variations between different datasets and genomes, RawHash2 calculates a weighted sum of multiple features that can impact the mapping decision. To achieve this, RawHash2 calculates normalized ratios of several metrics based on mapping quality and chain scores. These metrics are (i) the ratio of the mapping quality to a sufficiently high mapping quality (i.e., 30), (ii) mapping quality ratio between the best chain and the mean quality of all chains, and (iii) the ratio of the chain score between the best and the mean score of all chains. These ratios are combined into a weighted sum as follows: $w_{\text{sum}}=\sum_{i=1}r_{i}\times w_{i}$, where *r_i_* is a ratio of a particular metric, and *w_i_* is the weight assigned for that particular metric. The weighted sum, *w*_sum_, is compared against a predefined threshold value to decide if a read is considered to be mapped. RawHash2 maps a read if the weighted sum exceeds the threshold. Such a weighted sum approach allows RawHash2 to adaptively consider multiple aspects of the data and eliminates the potential effect of the ordering of these checks to achieve improved mapping accuracy while maintaining computational efficiency.

### 2.5 Minimizer sketching

RawHash provides the opportunity to integrate the existing hash-based sketching techniques such as minimizers (Roberts *et al.* 2004, Li 2018) for (i) reduced storage requirements of index in disk and memory and (ii) faster mapping due to fewer seed queries and hits.

To reduce the storage requirements of storing seeds in raw signals and due to their widespread application, RawHash2 integrates minimizers in two steps. First, RawHash2 generates hash values for seeds in both the reference genome and the raw signal. Second, within each window comprising *w* hash values, the minimum hash value is selected as the minimizer. These minimizer hash values can be used to find similarities using hash tables (similar to RawHash that uses hash values of all k-mers) while significantly reducing the number of hash values that need to be stored and queried during the mapping process as opposed to storing all k-mers.

### 2.6 Support for new data formats and flow cells

To enable better and faster adoption, RawHash2 incorporates support for (i) recent data formats for storing raw signals, namely POD5 and SLOW5 (Gamaarachchi *et al.* 2022) as well as the existing FAST5 format, and (ii) the latest flow cell versions due to two main reasons. First, transitioning from the FAST5 to the POD5 file format is crucial for broad adoption, as POD5 is the new standard file format introduced by Oxford Nanopore Technologies (ONT). Second, integrating the newer flow cell versions is challenging as it requires optimization of parameters involved in mapping decisions as well as segmentation. RawHash2 enables mapping the raw signals from R10.4 flow cells by optimizing the segmentation parameters for R10.4 and adjusting the scoring parameters involved in chaining settings to enable accurate mapping for R10.4 flow cells.

## 3 Results

### 3.1 Evaluation methodology

We implement the improvements we propose in RawHash2 directly on the RawHash implementation. Similar to RawHash, RawHash2 provides the mapping information using a standard pairwise mapping format (PAF).

We compare RawHash2 with the state-of-the-art works UNCALLED (Kovaka *et al.* 2021), Sigmap (Zhang *et al.* 2021), RawHash (Firtina *et al.* 2023a) in terms of throughput, accuracy, and the number of bases that need to be processed before stopping the sequencing of a read to estimate the benefits in sequencing time and cost. We provide the release versions of these tools in Supplementary Table S9. For throughput, we calculate the number of bases that each tool can process per second per CPU thread, which is essential to determine if a calculation in a single thread is at least as fast as the speed of sequencing from a single nanopore (i.e., single pore). In many commonly used nanopore sequencers, a nucleic acid molecule passes through a pore at around 450 bases and 400 per second with sampling rates of 4 KHz and 5 KHz for DNA in R9.4.1 and R10.4.1, respectively (Kovaka *et al.* 2021, Sam Kovaka *et al.* 2024). Since each read is mapped using a single thread for all tools, the throughput calculation is not affected by the number of threads available to these tools. Rather, this throughput calculation shows how many pores a single thread can process and how many CPU threads are needed to process the entire flow cell with many pores (e.g., 512 pores in a MinION flow cell). To show these results, we calculate (i) the number of pores that a single thread can process by dividing throughput by the number of bases sequenced per second per single pore and (ii) the number of threads needed to cover the entire flow cell.

For accuracy, we analyze three use cases: (i) read mapping, (ii) contamination analysis, and (iii) relative abundance estimation. To identify the correct mappings, we generate the ground truth mapping output in PAF by mapping the basecalled sequences of corresponding raw signals to their reference genomes using minimap2 (Li 2018). We use UNCALLED pafstats to compare the mapping output from each tool with their corresponding ground truth mapping output to calculate precision (P=TP/(TP+FP)), recall (R=TP/(TP+FN)), and F1 (F1=2×(P×R)/(P+R)) values, similar to RawHash (Firtina *et al.* 2023a). For read mapping, we compare the tools in terms of their precision, recall, and F-1 scores. For contamination analysis, the goal is to identify if a particular sample is contaminated with a certain genome (or set of genomes), which makes the precision metric more important for such a use case. For this use case, we compare the tools in terms of their precision in the main paper and show the full results (i.e., precision, recall, and F1) in Supplementary Table S1. For relative abundance estimation, we calculate the abundance ratio of each genome based on the ratio of reads mapped to a particular genome compared to all read mappings. We calculate the Euclidean distance of each estimation to the ground truth estimations generated based on minimap2 mappings of corresponding basecalled reads. We estimate the relative abundances based on the number of mapped reads rather than the number of mapped bases as we identify that larger genomes usually require sequencing a larger number of bases to map a read, which can lead to skewed estimations toward larger genomes.

To estimate the benefits in sequencing time and the cost per read, we identify the average sequencing length before making the mapping decision for a read. For all of our analyses, we use the default parameters of each tool as we show in Supplementary Table S7. Supplementary Table S6 shows the real dataset details we use in our evaluation, including more details about sequencing run settings and flow cell versions (i.e., R9.4.1 and R10.4). For all the datasets except D7, we use already basecalled reads available with the raw electrical signals. For the D7 dataset, we basecall the raw signals using the Dorado basecaller. Although RawHash2 does not use the minimizer sketching technique by default to achieve the maximum accuracy, we evaluate the benefits of minimizers in RawHash2, which we refer to as RawHash2-Minimizer. Since the evaluated versions of UNCALLED, Sigmap, and RawHash do not provide the support for R10.4 dataset, we show the corresponding results with the R10.4 dataset without comparing to these tools. When comparing RawHash2 to other tools we always use FAST5 files containing raw signals from R9.4 flow cells on an isolated machine and SSD. We use AMD EPYC 7742 processor at 2.26 GHz to run the tools. We use 32 threads for all the tools.

### 3.2 Throughput

**Figure 2.**
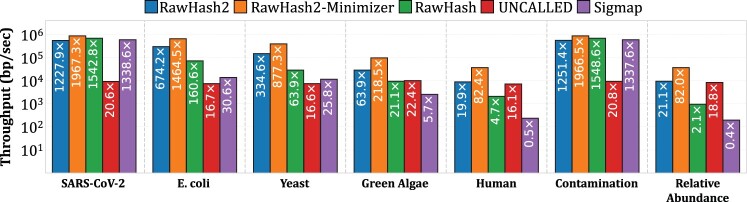
Throughput of each tool. Values inside the bars show how many nanopores (i.e., pores) that a single CPU thread can process.

Figure 2 shows the results for (i) throughput per single CPU thread and (ii) number of pores that a single CPU thread can analyze as annotated by the values inside the bars. We make three key observations. First, we find that RawHash2 provides average throughput 26.5×, 19.2×, and 4.0× better than UNCALLED, Sigmap, and RawHash, respectively. Such a speedup, specifically over the earlier work RawHash, is achieved by reducing the workload of chaining with the unique and accurate hash values using the new quantization mechanism and the filtering technique (see the filtering ratios in Supplementary Table S5). Second, we find that RawHash2-Minimizer enables reducing the computational requirements for mapping raw signals and enables improving the average throughput by 2.5× compared to RawHash2, while the other computational resources, such as the peak memory usage and CPU time in both indexing and mapping, and the mean time spent per read are also significantly reduced as shown in Supplementary Table S3 and Supplementary Fig. S3. Third, RawHash2-Minimizer requires *at most* 7 threads for analyzing the entire flowcell for any evaluated dataset, while RawHash2 requires at most 2 threads for smaller genomes and 9–26 threads for Green Algae and human. This shows that RawHash2 and RawHash2-Minimizer can reduce computational requirements and energy consumption significantly compared to 28 threads required, on average, regardless of the genome size for UNCALLED, which is critical for portable sequencing. We conclude that RawHash2 and RawHash2-Minimizer significantly reduce the computational overhead of mapping raw signals to reference genomes, enabling better scalability to even larger genomes.

### 3.3 Accuracy

Table 1 shows the accuracy results for read mapping, contamination analysis, and relative abundance estimation based on their corresponding most relevant accuracy metrics (results with all metrics are shown in Supplementary Table S1 and Supplementary Fig. S2). We make two key observations. First, we find that RawHash2 provides (i) the best accuracy in terms of the F1 score in all datasets for read mapping, (ii) the best precision for contamination analysis, and (iii) the most accurate relative abundance estimation. This is mainly achieved because (i) the adaptive quantization enables finding more accurate mapping positions while substantially reducing the false seed hits due to less precise quantization in RawHash, and (ii) the more sensitive chaining implementation with penalty scores can identify the correct mappings more accurately. Second, RawHash2-Minimizer provides mapping accuracy similar to that of RawHash2 with an exception for the human genome and better accuracy than RawHash, providing substantially better performance results as discussed in Section 3.2. Such an accuracy-performance trade-off puts RawHash2-Minimizer in an important position when a slight drop in accuracy can be tolerated for a particular use case when a substantially better throughput is needed. For the relatively lower accuracy that RawHash2 and RawHash2-Minimizer achieve compared to minimap2, we believe the accuracy gap is due to the increased difficulty in distinguishing the chain with the correct mapping position among many chains with similar quality scores, potentially due to the false seed matches in repetitive regions. Although our in-house evaluation shows that accuracy can substantially be improved further by enabling the correct chains to be distinguished more accurately than the incorrect chains with more sensitive quantization parameters, this comes with increased performance costs due to increased seed matches and chaining calculations. Future work can focus on designing more sensitive filters to improve the accuracy for larger and repetitive genomes by eliminating seed matches from such false regions. We conclude that RawHash2 is the most accurate tool regardless of the genome size, while the minimizer sketching technique in RawHash2-Minimizer can provide better accuracy than RawHash and on-par accuracy to all other tools while providing the best overall performance.

**Table 1.** Accuracy.^a^

Dataset	Metric	RH2	RH2-Min.	RH	UNCALLED	Sigmap
SARS-CoV-2	F1	**0.9867**	0.9691	0.9252	0.9725	0.7112
*E.coli*	F1	**0.9748**	0.9631	0.9280	0.9731	0.9670
Yeast	F1	**0.9602**	0.9472	0.9060	0.9407	0.9469
Green algae	F1	**0.9351**	0.9191	0.8114	0.8277	0.9350
Human	F1	**0.7599**	0.6699	0.5574	0.3197	0.3269
Contamination	Precision	**0.9595**	0.9424	0.8702	0.9378	0.7856
Rel. abundance	Distance	**0.2678**	0.4243	0.4385	0.6812	0.5430

a Best results are **highlighted**.

### 3.4 Sequencing time and cost

Table 2 shows the average sequencing lengths in terms of bases and chunks that each tool needs to process before stopping the sequencing process of a read. Processing fewer bases can significantly help reduce the overall sequencing time and potentially the cost spent for each read by enabling better utilization of nanopores without sequencing the reads unnecessarily. We make three key observations. First, RawHash2 reduces the average sequencing length by 1.9× compared to RawHash mainly due to the improvements in mapping accuracy, which enables making quick decisions without using longer sequences. Second, as the genome size increases, RawHash2 provides the smallest average sequencing lengths compared to all tools. Third, when the average length of sequencing is combined with other important metrics such as mapping accuracy in terms of F1 score and throughput, RawHash2 provides the best trade-off in terms of all these three metrics for all datasets as shown in Supplementary Fig. S4. We conclude that RawHash2 is the best tool for longer genomes to reduce the sequencing time and cost per read as it provides the smallest average sequencing lengths, while UNCALLED is the best tool for shorter genomes.

**Table 2.** Average length of sequencing per read.^a^

Dataset	RH2	RH2-Min.	RH	UNCALLED	Sigmap
SARS-CoV-2	443.92	460.85	513.95	**184.51**	452.38
*E.coli*	851.31	1030.74	1376.14	**580.52**	950.03
Yeast	**1147.66**	1395.87	2565.09	1233.20	1862.69
Green algae	**1385.59**	1713.46	4760.59	5300.15	2591.16
Human	**2130.59**	2455.99	4773.58	6060.23	4680.50
Contamination	670.69	**667.89**	742.56	1582.63	927.82
Rel. abundance	**1024.28**	1182.04	1669.46	2158.50	1533.04

a Best results are **highlighted**.

### 3.5 Evaluating new file formats and R10.4

In Supplementary Tables S2 and S4, we show the results when using different file formats for storing raw signals (i.e., FAST5, POD5, and BLOW5) and R10.4, respectively. We make two key observations. First, we find that POD5 and SLOW5 significantly speed up total elapsed time compared to FAST5. These results indicate that a large portion of the overhead spent for reading from a file can be mitigated with approaches that can perform faster compression and decompression, as these signal files are mostly stored in a compressed form. Second, we find that RawHash2 can perform fast analysis with reasonable accuracy that can be useful for certain use cases (e.g., contamination analysis) when using raw signals from R10.4, although RawHash2 achieves lower accuracy with R10.4 than using R9.4. This is likely because (i) we use a k-mer model optimized for the R10.4.1 flow cell version rather than R10.4, and (ii) minimap2 can provide more accurate mapping due to improved accuracy of these basecalled reads. Future work can focus on generating a k-mer model specifically designed for R10.4 to generate more accurate results. We exclude the accuracy results for R10.4.1 as the number of events found for R10.4.1 is around 35% larger than that of R10.4, which leads to inaccurate mapping. We suspect that our segmentation algorithm and parameters are not optimized for R10.4.1. Our future work will focus on improving these segmentation parameters and techniques to achieve higher accuracy with R10.4.1 as well as RNA sequencing data. We believe this can be achieved because RawHash2 (i) is highly flexible to change all the parameters corresponding to segmentation and (ii) can map accurately without requiring long sequencing lengths (Table 2), which can mainly be useful for RNA read sets. We conclude that RawHash2 can provide accurate and fast analysis when using the recent features released by ONT.

## 4 Conclusion

We introduce RawHash2, a tool that provides substantial improvements over the previous state-of-the-art mechanism RawHash. We make five key improvements over RawHash: (i) more sensitive quantization and chaining, (ii) reduced seed hits with filtering mechanisms, (iii) more accurate mapping decisions with weighted decisions, (iv) the first minimizer sketching technique for raw signals, and (v) integration of the recent features from ONT. We find that RawHash2 provides substantial improvements in throughput and accuracy over RawHash. We conclude that RawHash2, overall, is the best tool for mapping raw signals due to its combined benefits in throughput, accuracy, and reduced sequencing time and cost per read compared to the existing mechanisms, especially for longer genomes.

## Supplementary Material

btae478_Supplementary_Data
