# Supplementary material for: RawHash2: mapping raw nanopore signals using hash-based seeding and adaptive quantization
Source: Bioinformatics. 2024 Jul 30;40(8):btae478. doi: 10.1093/bioinformatics/btae478 (PMC11333567; doi:10.1093/bioinformatics/btae478)
Supplement: btae478_Supplementary_Data [file btae478_supplementary_data.pdf]

Supplementary Material for RawHash2: Mapping Raw Nanopore Signals Using Hash-Based Seeding and Adaptive Quantization

A. RawHash Overview

RawHash2 builds improvements over RawHash [1], a mechanism that provides the first hash-based similarity identification between a raw signal and a reference genome accurately and quickly. We show the overview of RawHash in Supplementary Figure S1. RawHash has four key steps. First, to generate sequences of signals that can be compared to each other, RawHash generates signals of k-mers, called *events*, from both a reference genome and raw signals. To generate events from reference genomes, it uses a lookup table, called *k-mer model*, that provides the expected signal value (i.e., event value) as a floating value for each possible k-mer where k is usually 6 or 9, depending on the flow cell version. To identify events (i.e., k-mers) in raw signals, RawHash performs a segmentation technique to detect the abrupt changes in signals, which enables identifying the regions in signals generated when sequencing a particular k-mer. RawHash uses the average value of signals within the same region as an event value. Due to the variations and noise in nanopore sequencing, event values can slightly differ from each other although they correspond to the same k-mer, making it challenging to directly match the event values to each other to identify matching k-mers between a reference genome and raw signals.

Second, to mitigate this noise issue, RawHash quantizes the event values such that slightly different event values can be quantized into the same value to enable direct matching of quantized event values between a reference genome and raw signals.

Third, to reduce the number of potential matches without reducing accuracy, RawHash concatenates the quantized event values of consecutive events (i.e., consecutive k-mers) and generates a hash value from these concatenated values.

Fourth, for the reference genome, these hash values are stored in a hash table along with their position information, which is usually known as the indexing step in read mapping. RawHash uses the hash values of raw signals to query the previously constructed hash table to identify matching hash values, known as *seed hits*, between a reference genome and a raw signal, which is then followed by chaining and mapping based on the seed hits.

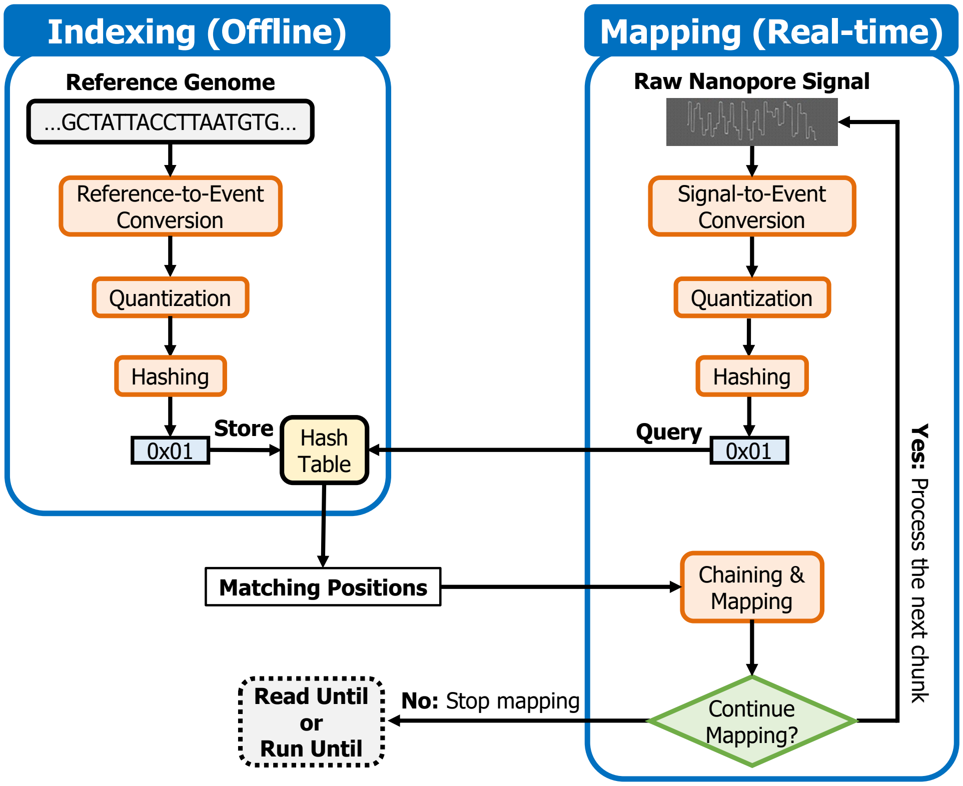

Figure S1: Overview of RawHash.

B. Accuracy

B.1. Read Mapping Accuracy

In Supplementary Table S1, we show the read mapping accuracy in all metrics (i.e., F1, Precision, and Recall) for all datasets. In Figure S2, we show the same results as reported in Supplementary Table S1 for visualizing the comparisons between tools and the trade-offs between each accuracy metric in all datasets.

Table S1: Read mapping accuracy in all metrics: F1, Precision, and Recall.

Dataset	Metric	RH2	RH2-Min.	RH	UNCALLED	Sigmap
SARS-CoV-2	F1	**0.9867**	0.9691	0.9252	0.9725	0.7112
	Precision	**0.9939**	0.9868	0.9832	0.9547	0.9929
	Recall	0.9796	0.9521	0.8736	**0.9910**	0.5540
E. coli	F1	**0.9748**	0.9631	0.9280	0.9731	0.9670
	Precision	**0.9904**	0.9865	0.9563	0.9817	0.9842
	Recall	0.9597	0.9408	0.9014	**0.9647**	0.9504
Yeast	F1	**0.9602**	0.9472	0.9060	0.9407	0.9469
	Precision	0.9553	0.9561	0.9852	0.9442	**0.9857**
	Recall	**0.9652**	0.9385	0.8387	0.9372	0.9111
Green Algae	F1	**0.9351**	0.9191	0.8114	0.8277	0.9350
	Precision	0.9284	0.9280	0.9652	0.8843	**0.9743**
	Recall	**0.9418**	0.9104	0.6999	0.7779	0.8987
Human	F1	**0.7599**	0.6699	0.5574	0.3197	0.3269
	Precision	0.8675	0.8511	**0.8943**	0.4868	0.4288
	Recall	**0.6760**	0.5523	0.4049	0.2380	0.2642
Contamination	F1	0.9614	0.9317	0.8718	**0.9637**	0.6498
	Precision	**0.9595**	0.9424	0.8702	0.9378	0.7856
	Recall	0.9632	0.9212	0.8736	**0.9910**	0.5540
Rel. Abundance	F1	**0.4659**	0.3375	0.3045	0.1249	0.2443
	Precision	**0.4623**	0.3347	0.3018	0.1226	0.2366
	Recall	**0.4695**	0.3404	0.3071	0.1273	0.2525

Best results are **highlighted**.

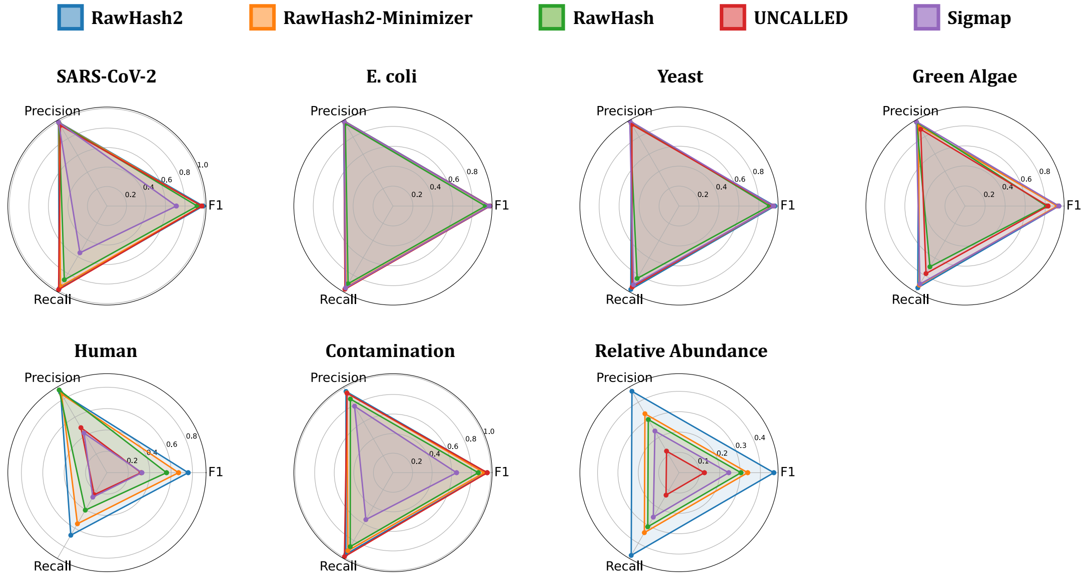

Figure S2: Read mapping accuracy results in terms of F1 score, precision, and recall across different datasets. The dotted triangles show the best possible results, where each edge shows the best result for its corresponding metric.

B.2. R10.4 Accuracy and Performance

In Supplementary Table S2, we show the accuracy and performance results in terms of throughput and mean time spent per read when using R10.4 flow cells. For comparison purposes between R10.4 and R9.4, we include the results from R9.4 flow cells for *E. coli*. We do not show the R9.4 results for *S. aureus*, since we do not have raw signals from the same sample for this dataset.

Table S2: Accuracy and performance results when using R10.4 and R9.4 datasets

Flow Cell		RH2	RH2-Min.
**Read Mapping Accuracy (E. coli)**			
R9.4	F1	0.9748	0.9631
	Precision	0.9904	0.9865
	Recall	0.9597	0.9408
R10.4	F1	0.8960	0.8389
	Precision	0.9506	0.9325
	Recall	0.8473	0.7623
**Read Mapping Accuracy (S. aureus)**			
R10.4	F1	0.7749	0.6778
	Precision	0.8649	0.8167
	Recall	0.7018	0.5793
**Performance (E. coli)**			
R9.4	Throughput [bp/sec]	303,382.45	659,013.57
	Mean time per read [ms]	2.161	1.099
R10.4	Throughput [bp/sec]	175,351.94	480,471.75
	Mean time per read [ms]	6.598	2.505
**Performance (S. aureus)**			
R10.4	Throughput [bp/sec]	256,680.4	617,308.7
	Mean time per read [ms]	5.478	2.243

C. Performance

C.1. Runtime, Peak Memory Usage, and Throughput

Supplementary Table S3 shows the computational resources required by each tool during the indexing and mapping steps. To measure the required computational resources, we collect CPU time and peak memory usage of each tool for all the datasets. To collect these results, we use `time -v` command in Linux. CPU time shows the total user and system time. Peak memory usage shows the maximum resident set size in the main memory that the application requires to complete its task. To measure the CPU threads needed for analyzing the entire MinION Flowcell with 512 pores, we divide 512 with the number of pores that a single thread can process (as shown with the values inside the bars in Figure 2) and round up the values to provide the maximum number of threads needed.

Table S3: Computational resources required in the indexing step of each tool.

Dataset	RH2	RH2-Min.	RH	UNCALLED	Sigmap
Indexing CPU Time (sec)					
SARS-CoV-2	0.12	0.06	0.16	8.40	**0.02**
E. coli	2.48	**1.61**	2.56	10.57	8.86
Yeast	4.56	**3.02**	4.44	16.40	25.29
Green Algae	27.60	**17.73**	24.51	213.13	420.25
Human	1,093.56	**588.30**	809.08	3,496.76	41,993.26
Contamination	0.13	0.06	0.15	8.38	**0.03**
Rel. Abundance	747.74	**468.14**	751.67	3,666.14	36,216.87
Indexing Peak Memory (GB)					
SARS-CoV-2	**0.01**	**0.01**	**0.01**	0.06	**0.01**
E. coli	0.35	0.19	0.35	**0.11**	0.40
Yeast	0.75	0.39	0.76	**0.30**	1.04
Green Algae	5.11	**2.60**	5.33	11.94	8.63
Human	80.75	**40.59**	83.09	48.43	227.77
Contamination	**0.01**	**0.01**	**0.01**	0.06	**0.01**
Rel. Abundance	152.59	75.62	152.84	**47.80**	238.32
Mapping CPU Time (sec)					
SARS-CoV-2	1,705.43	**1,227.05**	1,539.64	29,282.90	1,413.32
E. coli	1,296.34	**787.49**	7,453.21	28,767.58	22,923.09
Yeast	545.77	**246.37**	4,145.38	7,181.44	7,146.32
Green Algae	2,135.83	**657.63**	22,103.03	12,593.01	26,778.44
Human	100,947.58	**21,860.05**	1,825,061.23	245,128.15	6,101,179.89
Contamination	3,783.69	**2,332.28**	3,480.43	234,199.60	3,011.78
Rel. Abundance	250,076.90	**62,477.76**	4,551,349.79	569,824.13	15,178,633.11
Mapping Peak Memory (GB)					
SARS-CoV-2	4.15	4.16	4.20	**0.17**	28.26
E. coli	4.13	4.03	4.18	**0.50**	111.12
Yeast	4.38	4.12	4.37	**0.36**	14.66
Green Algae	6.11	4.98	11.77	**0.78**	29.18
Human	48.75	25.04	52.43	**10.62**	311.94
Contamination	4.16	4.14	4.17	**0.62**	111.70
Rel. Abundance	49.14	25.82	54.89	**8.99**	486.63
Mapping Throughput (bp/sec)					
SARS-CoV-2	552,561.25	**885,263.48**	694,274.92	9,260.31	602,380.96
E. coli	303,382.45	**659,013.57**	72,281.32	7,515.76	13,750.97
Yeast	150,547.61	**394,766.80**	28,757.15	7,471.48	11,624.82
Green Algae	28,742.46	**98,323.70**	9,488.79	10,069.41	2,569.89
Human	8,968.78	**37,086.38**	2,099.35	7,225.67	236.45
Contamination	563,129.81	**884,929.30**	696,873.20	9,343.95	601,936.49
Rel. Abundance	9,501.37	**36,919.79**	962.79	8,437.70	196.48
CPU Threads Needed for the entire MinION Flowcell (512 pores)					
SARS-CoV-2	**1**	**1**	**1**	25	**1**
E. coli	**1**	**1**	4	31	17
Yeast	2	**1**	9	31	20
Green Algae	9	**3**	25	23	90
Human	26	**7**	110	32	975
Contamination	**1**	**1**	**1**	25	**1**
Rel. Abundance	25	**7**	240	28	1173

Best results are **highlighted**.

C.2. Impact of Different File Formats on Performance

Supplementary Table S4 shows the overall execution time when using different raw signal file formats: FAST5, POD5, and BLOW5 [2]. To evaluate the direct impact of these formats, we run RawHash2 (RH2) and RawHash2-Minimizer (RH2-Min.) 1) using a single thread (i.e., single thread for the entire execution including *both* file IO and mapping), 2) using an isolated SSD on a PCI-e interface, 3) using the same compression type (i.e., zstd) for all file formats, and 4) clearing the disk cache before each execution. When using a single thread, we confirm that the underlying libraries for FAST5, POD5, and BLOW5 are not aggressively using more threads than what is allocated to them, as the thread utilization is reported as 0.99 (i.e., 99%) by the `time -v` command for the entire execution.

We note that even if we use multiple threads when running RawHash2, the file IO step (i.e., reading from or writing to a file) always uses a single thread and is overlapped with the mapping step (i.e., either the read or write operation is run in parallel together with the mapping step by using one thread where the mapping step takes rest of the allocated threads). The design is due to the pipelining implementation strategy we adopt, similar to the minimap2 implementation [19]. We note that if RawHash2 is run using a single thread, none of these steps overlap with each other, and they run sequentially using only one thread, which is our evaluation setting we show in Supplementary Table S4.

Table S4: Comparison of overall execution time when using different file formats in RawHash2 in a single-threaded mode.

Tool	*E. coli*	*Yeast*
	Elapsed Time (mm:ss)	
RH2-FAST5	19:27	08:35
RH2-POD5	16:55	07:33
RH2-BLOW5	17:32	07:38
RH2-Min.-FAST5	12:13	03:56
RH2-Min.-POD5	09:42	02:56
RH2-Min.-BLOW5	10:16	03:02

C.3. Mapping Time per Read

Supplementary Figure S3 shows the average mapping time that each tool spends per read for all the datasets we evaluate. The mapping times spent per read are provided by each tool as PAF output with the `mt` tag. We use these reported values to calculate the average mapping time across all reads reported in their corresponding PAF files.

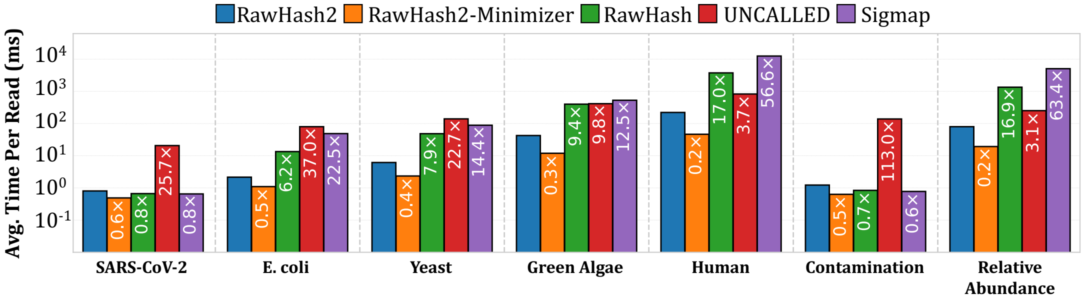

Figure S3: Average time spent per read by each tool in real-time. Values inside the bars show the speedups that RawHash2 provides over other tools in each dataset.

C.4. Combined benefits of performance, accuracy, and average sequencing length

Supplementary Figure S4 shows the combined results of each tool in terms of throughput, F-1 Score (i.e., accuracy), and average sequencing length for each dataset. The dotted lines in each triangle show the ideal combined result. Each edge of the triangle shows the best result for the corresponding metric, as shown in the figure.

For the edge that shows the F-1 score, the best point is 1.0. All tools have F-1 scores between 0 and 1, as shown in Table 1. For the other two edges, which show throughput and average sequencing length, the best result is determined based on the highest result we observe for that dataset. We adjust all other results using these highest results so that the adjusted throughput and average sequencing length values are always between 0 and 1.

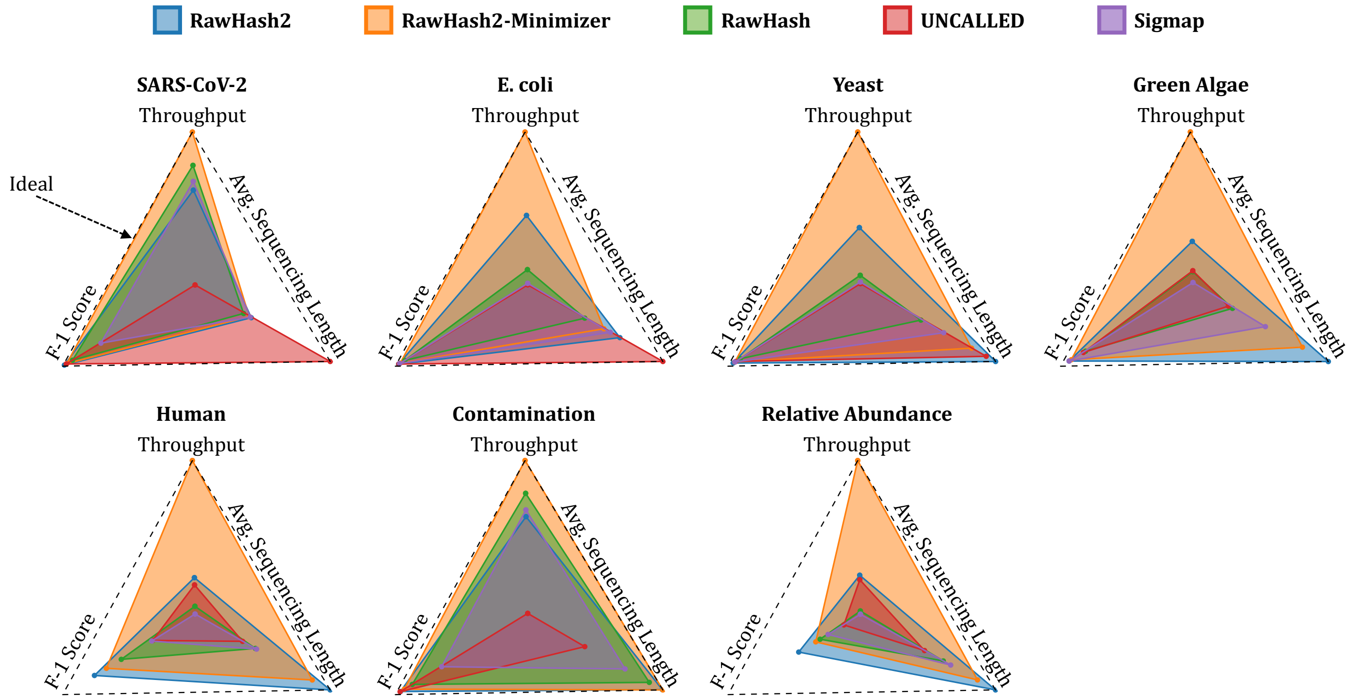

Figure S4: Combined results in terms of throughput, F-1 score (i.e., accuracy), and average sequencing length across different datasets. The dotted triangles show the best possible results, where each edge shows the best result for its corresponding metric.

C.5. Ratio of Filtered Seeds from Frequency Filter

Supplementary Table S5 shows the ratio of seed hits filtered out by the frequency filtered in RawHash2. We calculate these ratios in three steps. First, for each seed (i.e., a hash value that RawHash2 constructs from raw signals), we perform a query to the hash table that is used as an index. If the hash value exists, the table returns a list of genomic regions that share the same hash value. Each region counts as a seed hit, and the list length indicates the number of seed hits. Second, for all seeds generated from raw signals, we count 1) the overall number of seed hits and 2) the number of seed hits filtered out by frequency filter. We note that if the list length (i.e., number of seed hits) returned after querying a particular seed is above a certain threshold (defined by our frequency filter), all seed hits within the same list are filtered out. Third, we calculate the ratio of filtered seed hits to the total seed hits and report these ratios in Supplementary Table S5.

Table S5: Ratio of filtered seed hits from frequency filter.

Dataset	Average Filtered Ratio
SARS-CoV-2	0.0627
E. coli	0.5505
Yeast	0.5356
Green Algae	0.8106
Human	0.5104
E. coli (R10.4)	0.6895
S. aureus (R10.4)	0.6003

D. Configuration

D.1. Datasets

In Supplementary Table S6 we show the details of the datasets used in our evaluation and their corresponding sequencing run settings. The *Basecaller Model* column shows the details about the basecaller model and the version we use. Except for the D7 dataset, all other datasets include the basecalled sequences within their corresponding FAST5 files or the corresponding accession numbers available at NCBI. We provide the scripts to extract these basecalled sequences on the GitHub page of RawHash2. For the D7 dataset, we provide the necessary commands to run dorado for basecalling on the GitHub page.

Table S6: Details of datasets used in our evaluation.

	Organism	Device Type	Flow Cell Type	Transloc. Speed	Sampling Frequency	Basecaller Model	Reads (#)	Bases (#)	SRA Accession	Reference Genome	Genome Size
Read Mapping											
D1	*SARS-CoV-2*	MinION	R9.4.1 e8 (FLO-MIN106)	450	4000	Guppy HAC v3.2.6	1,382,016	594M	CADDE Centre	GCF_009858895.2	29,903
D2	*E. coli*	GridION	R9.4.1 e8 (FLO-MIN106)	450	4000	Guppy HAC v5.0.12	353,317	2,365M	ERR9127551	GCA_000007445.1	5M
D3	*Yeast*	MinION	R9.4.1 e8 (FLO-MIN106)	450	4000	Albacore v2.1.7	49,989	380M	SRR8648503	GCA_000146045.2	12M
D4	*Green Algae*	PromethION	R9.4.1 e8 (FLO-PRO002)	450	4000	Albacore v2.3.1	29,933	609M	ERR3237140	GCF_000002595.2	111M
D5	*Human*	MinION	R9.4.1 e8 (FLO-MIN106)	450	4000	Guppy Flip-Flop v2.3.8	269,507	1,584M	FAB42260	T2T-CHM13 (v2)	3,117M
D6	*E. coli*	GridION	R10.4 e8.1 (FLO-MIN112)	450	4000	Guppy HAC v5.0.16	1,172,775	6,123M	ERR9127552	GCA_000007445.1	5M
D7	*S. aureus*	GridION	R10.4 e8.1 (FLO-MIN112)	450	4000	Dorado SUP v0.5.3	407,727	1,281M	SRR21386013	GCF_000144955.2	2.8M
Contamination Analysis											
D1 and D5							1,651,523	2,178M	D1 and D5	D1	29,903
Relative Abundance Estimation											
D1-D5							2,084,762	5,531M	D1-D5	D1-D5	3,246M

Multiple dataset numbers in contamination analysis and relative abundance estimation show the combined datasets.
D1-D5 datasets are from R9.4, and D6 and D7 are from R10.4. Human reads are from Nanopore WGS.
Base counts in millions (M).

D.2. Parameters

In Supplementary Table S7, we show the parameters of each tool for each dataset. In Supplementary Table S8, we show the details of the preset values that RawHash2 sets in Supplementary Table S7. For UNCALLED [3], Sigmap [4], and minimap2 [5], we use the same parameter setting for all datasets. For the sake of simplicity, we only show the parameters we explicitly set in each tool. For the descriptions of all the other parameters, we refer to the help message that each tool generates, including RawHash2.

Table S7: Parameters we use in our evaluation for each tool and dataset in mapping.

Tool	*Contamination*	*SARS-CoV-2*	*E. coli* (R9.4)	*Yeast*	*Green Algae*	*Human*	*Rel. Abundance*	*E. coli* (R10.4)	*S. aureus* (R10.4)
RawHash2	-x viral −depletion -t 32	-x viral -t 32	-x sensitive -t 32	-x sensitive -t 32	-x sensitive -t 32	-x fast -t 32	-x fast -t 32	-x sensitive −r10 -t 32	-x sensitive −r10 -t 32
RawHash2-Minimizer	-x viral -w3 −depletion -t 32	-x viral -w3 -t 32	-x sensitive -w3 -t 32	-x sensitive -w3 -t 32	-x sensitive -w3 -t 32	-x fast -w3 -t 32	-x fast -w3 -t 32	-x sensitive −r10 -w3 -t 32	-x sensitive −r10 -w3 -t 32
RawHash	-x viral -t 32	-x viral -t 32	-x sensitive -t 32	-x sensitive -t 32	-x fast -t 32	-x fast -t 32	-x fast -t 32	NA	NA
UNCALLED				map -t 32				NA	NA
Sigmap				-m -t 32				NA	NA
Minimap2					-x map-ont -t 32				

Table S8: Corresponding parameters of presets (-x) in RawHash2.

**Preset**	**Corresponding parameters**	Usage
viral	-e 6 -q 4 −max-chunks 5 −bw 100 −max-target-gap 500 −max-target-gap 500 −min-score 10 −chain-gap-scale 1.2 −chain-skip-scale 0.3	Viral genomes
sensitive	-e 8 -q 4 −fine-range 0.4	Small genomes (i.e., < 500M bases)
fast	-e 8 -q 4 −max-chunks 20	Large genomes (i.e., > 500M bases)
	**Other helper parameters**	
depletion	−best-chains 5 −min-mapq 10 −w-threshold 0.5 −min-anchors 2 −min-score 15 −chain-skip-scale 0	Contamination analysis
r10	-k9 −seg-window-length1 3 −seg-window-length2 6 −seg-threshold1 6.5 −seg-threshold2 4 −seg-peak-height 0.2 −chain-gap-scale 1.2	For R10.4 Flow Cells

D.3. Versions

Supplementary Table S9 shows the version and the link to these corresponding versions of each tool and library we use in our experiments and in RawHash2, respectively.

Table S9: Versions of each tool and library.

Tool	Version	Link to the Source Code
RawHash2	2.1	`https://github.com/CMU-SAFARI/RawHash/releases/tag/v2.1`
RawHash	1.0	`https://github.com/CMU-SAFARI/RawHash/releases/tag/v1.0`
UNCALLED	2.3	`https://github.com/skovaka/UNCALLED/releases/tag/v2.3`
Sigmap	0.1	`https://github.com/haowenz/sigmap/releases/tag/v0.1`
Minimap2	2.24	`https://github.com/lh3/minimap2/releases/tag/v2.24`
		Library versions
FAST5 (HDF5)	1.10	`https://github.com/HDFGroup/hdf5/tree/db30c2d`
POD5	0.2.2	`https://github.com/nanoporetech/pod5-file-format/releases/tag/0.3.10`
S/BLOW5	1.2.0-beta	`https://github.com/hasindu2008/slow5lib/tree/e0d0d0f`

Supplementary References

[1] C. Firtina *et al.*, "RawHash: enabling fast and accurate real-time analysis of raw nanopore signals for large genomes," *Bioinformatics*, vol. 39, no. Supplement_1, pp. i297–i307, Jun. 2023.
[2] H. Gamaarachchi *et al.*, "Fast nanopore sequencing data analysis with SLOW5," *Nat. Biotechnol.*, 2022.
[3] S. Kovaka *et al.*, "Targeted nanopore sequencing by real-time mapping of raw electrical signal with UNCALLED," *Nature Biotechnology*, vol. 39, no. 4, pp. 431–441, Apr. 2021.
[4] H. Zhang *et al.*, "Real-time mapping of nanopore raw signals," *Bioinformatics*, vol. 37, no. Supplement_1, pp. i477–i483, Jul. 2021.
[5] H. Li, "Minimap2: pairwise alignment for nucleotide sequences," *Bioinformatics*, vol. 34, no. 18, pp. 3094–3100, Sep. 2018.
